# Lung segmentectomy assisted by highly selective independent segmental ventilation: a series of three cases

**DOI:** 10.1186/s13019-021-01474-2

**Published:** 2021-04-15

**Authors:** Xiaoshun Shi, Jing Ye, Junyong Chen, Jianxue Zhai, Xiguang Liu, Di Lu, Zishi Lin, Zhen Ni, Hua Wu, Kaican Cai

**Affiliations:** 1 Department of Thoracic Surgery, Nanfang Hospital, Southern Medical University, No. 1838 North Guangzhou Avenue, Guangzhou, 510515 China; 2 Department of Anesthesiology, Nanfang Hospital, Southern Medical University, Guangzhou, 510515 China

**Keywords:** Segmentectomy, Highly selective independent segmental ventilation, VATS, Intersegmental plane identification

## Abstract

**Background:**

The identification of targeted intersegmental planes and resection with adequate surgical margins are among the crucial steps in anatomical pulmonary segmentectomy, and technical improvements are still needed.

**Case presentation:**

We reported three cases of intersegmental plane identification using highly selective independent segmental ventilation during segmentectomy. All cases required cooperation with an anesthesiologist who was able to perform segmental ventilation and double confirmation of segmental bronchus branches by the surgeon. The surgical procedure provides a direct visualization of spare segment inflation and saves time in deflation over the conventional residual segment inflation method.

**Conclusions:**

Highly selective independent segmental ventilation could be considered a suitable option for pulmonary intersegmental plane identification and could be universally used for lung segmentectomy.

## Background

Precise intersegmental plane identification is a crucial step in anatomical segmentectomy, which is a surgical procedure aimed at curing small and early lung cancer while preserving lung function. Previous approaches have mainly focused on the modification of medical dyes and ventilation techniques, such as direct injection of indocyanine green (ICG) [1] and methylene blue [2] into the bronchi of target pulmonary segments, indirect intravenous injection of ICG visualized by infrared thoracoscopy (IRT) [3], inflation-deflation methods [4], the selective jet injection method [5], oxygen instillation into affected segments with a butterfly needle [6], the slip knot bronchial ligation method, and the modified inflation-deflation method [7]. These techniques are demanding due to time for lung collapse, pharmaceutical materials, special cameras, and potential risk of air embolism [8]. Therefore, a simpler and efficient method of intersegmental plane identification is required. With the advancement of the anesthesiologic technique, one-lung ventilation is possible [9], reaching the segmental level [10]. With specialized instruments and proper intubation techniques, direct inflation of a lung segment is possible. We reported three successful cases of intersegmental plane identification assisted using highly selective independent segmental ventilation during thoracoscopic segmentectomy.

## Case presentation

### Patients and surgical procedures

Three patients, 52, 78, and 60 years old, were admitted because small nodules less than 3 cm in size were found on computed tomography between April 2018 and May 2018. All of the patients met the indications for pulmonary segmentectomy. The VATS procedures included the lateral basal and posterior basal segments of the right lower lung (Case 1: S9 + 10), the apical, posterior and anterior segments of the left upper lobe (Case 2: S1 + 2 + 3), and the apical and posterior segments of the left upper lobe (Case 3: S1 + 2). All segmentectomies were performed by single-direction thoracoscopic segmentectomy [11–14].

### Highly selective independent segmental ventilation

**Fig. 1:**
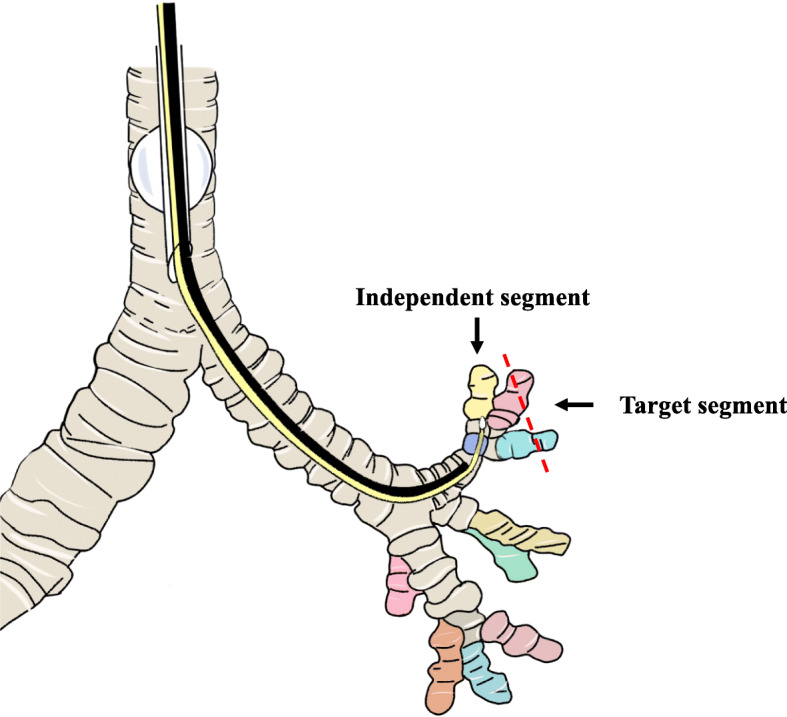
Schematic representation of highly selective independent segmental ventilation

The ventilation technique was approved by the Institutional Review Board and the Ethics Committee of Nanfang Hospital, Southern Medical University (IRB- 2012-156). A schematic representation of highly selective independent segmental ventilation is shown in Fig. 1.

### Preparation

Before anesthesia and ventilation, all of the patients were excluded from tracheal and bronchial variation by assessing the results of preoperative chest computed tomography and bronchoscopy. Anesthesia induction was performed by propofol sedation using target-controlled infusion, and then the use of a double-lumen endobronchial tube (DLT) was based on the bronchial diameter at the level of the sternal end of the clavicle. In general, we used 37 French gauges (Fr) with internal tube diameters (ITDs) of 4.7 mm or larger in most male patients and 32 Fr with ITDs of 3.5 mm or larger in most female patients. The use of the 5F Arndt pediatric endobronchial blocker (Cook Medical, Bloomington, USA) was based on product instructions. Notably, upon the completion of segment ventilation, the balloon endobronchial blocker must be deflated before removal.

### Step 1. Identification of independent segmental bronchial orifice

Two experienced anesthesiologists identified segmental bronchial orifices by fiberoptic bronchoscopy (FOB), advancing the endobronchial blocker to the orifice of independent segmental bronchus.

### Step 2. Intraoperative conformation of independent segmental bronchial orifice

**Fig. 2:**
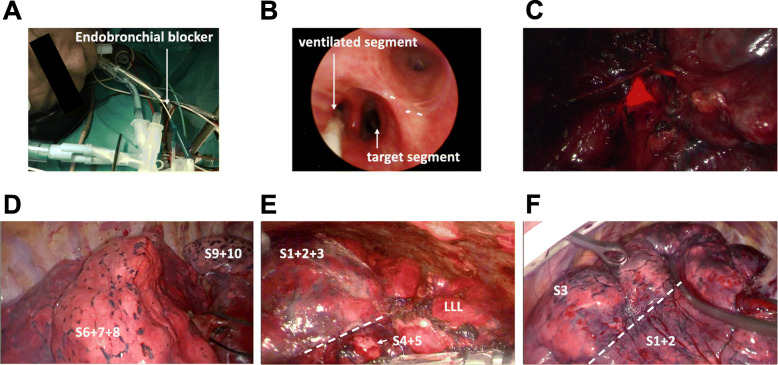
Illustration of lung segmentectomy assisted using highly selective independent segmental ventilation. **a** The insertion of the endobronchial blocker during general anesthesia. **b** Two experienced anesthesiologists performed the identification and visualization of the target segment. **c** The light of the fiberoptic bronchoscope at the orifice of the remaining pulmonary segment was visualized under VATS dark vision. The intersegmental plane appeared sufficiently slowly to easily visualize and manipulate the stapler across targeted segmental border in case 1 (**d**), case 2 with severe pleural adhesions (**e**) and case 3 (f). The remaining segment was inflated, but the remaining lobe remained deflated

Following the management of the target segmental structure (segmental vein, artery, and bronchus), intersegmental plane identification is required. Currently, the 5F Arndt pediatric endobronchial blocker enters the initial placement guided by FOB and is inserted into the orifice of the segmental bronchus of the independent-ventilated pulmonary segment (Fig. 2a-b).

### Step 3. Ventilation of independent pulmonary segment

After reconfirmation by the light of FOB under VATS dark vision (Fig. 2c) and the endo-stapler being clamped at the targeted segmental bronchi, 100% oxygen flowing at 3–5 L/min was ventilated by an anesthesiologist (Fig. 2d-f). The independent pulmonary segments were subsequently inflated without ventilation of the remaining pulmonary lobe.

After triple confirmation and marking of the intersegmental plane, an endo-stapler (Endo GIA™ 60 mm Articulating Medium/Thick Reload with Tri-Staple Technology Purple Cartridge, COVIDIEN Medical, USA) was introduced, and the segmental bronchi and the segmental border were divided. Lymph node dissection was then performed.

## Discussion and conclusions

We successfully performed 3 segmentectomies with highly selective independent segmental ventilation. There were no ventilation-associated complications after surgery. No segmentectomy-caused complications, such as air leaks, atelectasis or subcutaneous emphysema, were observed. The patients were discharged with an uneventful recovery course. A technique of quick and accurate identification of the plane of the lung segment is of particular importance in segmental resection. The conventional method is the inflation and deflation technique. The shortcoming of this method is that the identification of the target bronchus depends greatly on the subjective judgment of the surgeon, and the waiting time for deflation is long. Other identification techniques for the intersegmental plane have been described in previous studies. The technique of ultrafine fiber bronchoscopy with high-frequency bronchial ventilation (40 Hz, 2 ^kg/cm2^) at the target segment was proposed by Okada [15]. Misaki et al. visualized intersegmental junctions under infrared thoracoscopy when intravenous injection of indocyanine green (ICG; 3.0 mg/kg) was performed after ligation of the target segmental artery. Oh et al. reported a method of injection of ICG (25 mg dissolved in 50 mL of saline) at the distal peripheral bronchus after the target segment bronchus was controlled [16].

Highly selective lung segment ventilation is an anesthetic-surgical technique that combines the proficiency of bronchoscopy, ventilation and thoracoscopy under close cooperation between the surgeon and the anesthesiologist. The trinity of segmental bronchus identification by bronchoscopy, bronchoscopy light source under dark vision using VATS, and inflation of the remaining segment not affecting other pulmonary lobes ensures the safety of precise resection of the target lung segment. Compared with traditional ventilation, our highly selective independent segmental ventilation does not ventilate the remaining pulmonary lobe, avoiding the waiting time for lung collapse and not blocking the surgical field. Compared with traditional bronchoscope-guided high-frequency ventilation, our endobronchial blocker was inserted into the orifice of the independent segmental bronchus, which could effectively prevent potential damage to the endobronchial blocker and the bronchoscope. Therefore, this technology can accurately and quickly locate the stereoborder of the target lung segment, which is expected to shorten the operative time for lung segment resection and improve the efficiency of intersegmental plane identification.

The disadvantages of this technique include incurring the additional cost of the endobronchial blocker and the anesthesiologist’s experience in bronchoscopy beyond the trachea and main bronchus. However, the endobronchial blocker used in this technique is a conventional consumable. No costly special thoracoscopic equipment or dye injection is required. From anesthesiologists’ point of view, the idea of selective lobar blockade, selective segmental blockade or ventilation lives on in new, adaptive forms. With the increasing demand for ERAS in thoracic surgery, experienced anesthesiologists use this novel concept of lung isolation, which benefits our patients. Several different available bronchial blockers can be used to achieve lobar or segmental collapse; therefore, understanding the bronchoscopic anatomy of the pulmonary segment should be assured and is required in our hospital.

To our knowledge, this study is the first case series of pulmonary segmentectomy assisted by highly selective independent segmental ventilation. Because of the simplicity of this technique, we purpose that universal use could be feasible for resection of pulmonary segments, and the efficacy of this method should be estimated in a larger clinical trial.

## Data Availability

All images supporting the technique of this article are included within the article. We could provide surgical videos with patient consent.

## Abbreviations

DLT: Double-lumen endobronchial tube
FOB: Fiberoptic bronchoscopy
Fr: French gauges
ICG: Indocyanine green
IRT: Infrared thoracoscopy
ITD: Internal tube diameter
VATS: Video-assisted thoracoscopic surgery
